# Counteracting sarcopenia: the role of IGF-1 isoforms

**DOI:** 10.18632/aging.102027

**Published:** 2019-06-13

**Authors:** Antonio Musarò, Bianca Maria Scicchitano

**Affiliations:** 1 DAHFMO-Unit of Histology and Medical Embryology, Sapienza University of Rome, Laboratory affiliated to Istituto Pasteur Italia – Fondazione Cenci Bolognetti, Rome 00161, Italy; 2 Istituto di Istologia ed Embriologia, Università Cattolica del Sacro Cuore, Fondazione Policlinico Universitario “Agostino Gemelli”, IRCCS, Roma 00168, Italy

**Keywords:** sarcopenia, IGF-1, muscle, ROS, autophagy, NMJ

Sarcopenia, the age-related loss of muscle mass and strength, represents one of the main causes of impaired physical performance and reduced mobility. Thus, understanding the pathogenetic mechanisms of muscle wasting associated with aging has been the objective of numerous studies and represents an important first step for the development of therapeutic approaches [1,2].

Among growth factors, the insulin-like growth factor-1 (IGF-1) have been implicated in the control of skeletal muscle growth, differentiation, and regeneration and has emerged as a growth factor with a remarkably wide range of actions and a tremendous potential as a therapeutic factor in attenuating the atrophy and frailty associated with muscle ageing and diseases [3,4]. In the adult mammals, IGF-1 is principally synthesized in the liver, acting as a systemic growth factor; however, it is also produced in extrahepatic tissues, including skeletal muscle, where it mainly plays an autocrine/paracrine role. The IGF-1 protein is produced by different pre-pro-peptides, whereas two different promoters and differential splicing of the IGF-1 gene create several IGF-1 isoforms, which differ in the N-terminal signal-peptide (Class 1 or 2) and the C-terminal Extension peptide (E-peptide Ea or Eb) [3]. Given the conflicting and still unclear data on effects of different IGF-1 isoforms, a recent study investigated whether the muscle overexpression of either propeptides IGF-1Ea or IGF-1Eb isoform impacts sarcopenia and through which mechanisms each isoform acts [5]. Muscle restricted over-expression of both IGF-1Ea and IGF-1Eb isoforms did not induce any significant change in the circulating IGF-1 levels in young mice compared to age-matched wild type animals. Interestingly, consistent with the physiological decline of IGF-1 plasma levels during aging, a strong reduction of IGF-1 levels was observed in old wild type mice [5]. On the contrary, aged transgenic animals showed unchanged levels of circulating IGF-1 compared to young counterparts, thus resulting higher compared to old wild type mice. It is possible to speculate that IGF-1 isoforms, locally expressed, exert an indirect systemic effect, contributing to the maintenance of circulating IGF-1 levels during postnatal life. Indeed, skeletal muscle has recently been identified as an endocrine organ, able to produce and release cytokines and other peptides, such as the myokines, that act in paracrine, autocrine, or endocrine manner [6]. In this context, muscle can be also a source of circulating IGF-1, based on the evidences that IGF-1 is released from exercising muscle into the blood stream [6]. Thus, we can speculate that the reduction of circulating IGF-1 levels in aged wild type mice could be the result of the morpho-functional alterations occurred in muscle. Conversely, transgenic mice, guaranteeing the muscle expression levels of IGF-1 isoforms even at late post-natal life, preserve the capability of muscle to function as endocrine organ, thus contributing to maintain unaltered the circulating IGF-1 levels.

Of note, IGF-1Ea but not IGF-1Eb was able to promote a pronounced muscle hypertrophy and strength in both young and aged mice [5]. Nevertheless, beside the promotion of muscle growth, both IGF-1Ea and IGF-1Eb were able to counteract sarcopenia, negatively modulating the inflame-aging process and activating relevant pathways considered part of a molecular antiaging system, such as autophagy and PGC-1-mediated signaling, which alteration induces neuromuscular junction degeneration and precocious aging [5,7]. Of interest, and in some way paradoxically, the increased levels of IGF-1Ea, but not IGF-1Eb, was correlated with high ROS production [5]. This data suggests that ROS production is part of the promotion and maintenance of a functional hypertrophic phenotype, induced by IGF-1Ea, and supports the evidence that reactive oxygen species are not merely damaging agent but useful signaling molecules to regulate growth, proliferation, differentiation, and adaptation, at least within physiological concentration. Of note, the IGF-1Ea mice were able to minimize oxidative damage in senescent muscle up-regulating, through PGC1-α activation, NRF-2 protein, the master regulator of antioxidant defense [8] and Sirt-1, a factor involved in growth regulation, stress response, endocrine signaling, and extended lifespan.

**Figure 1:**
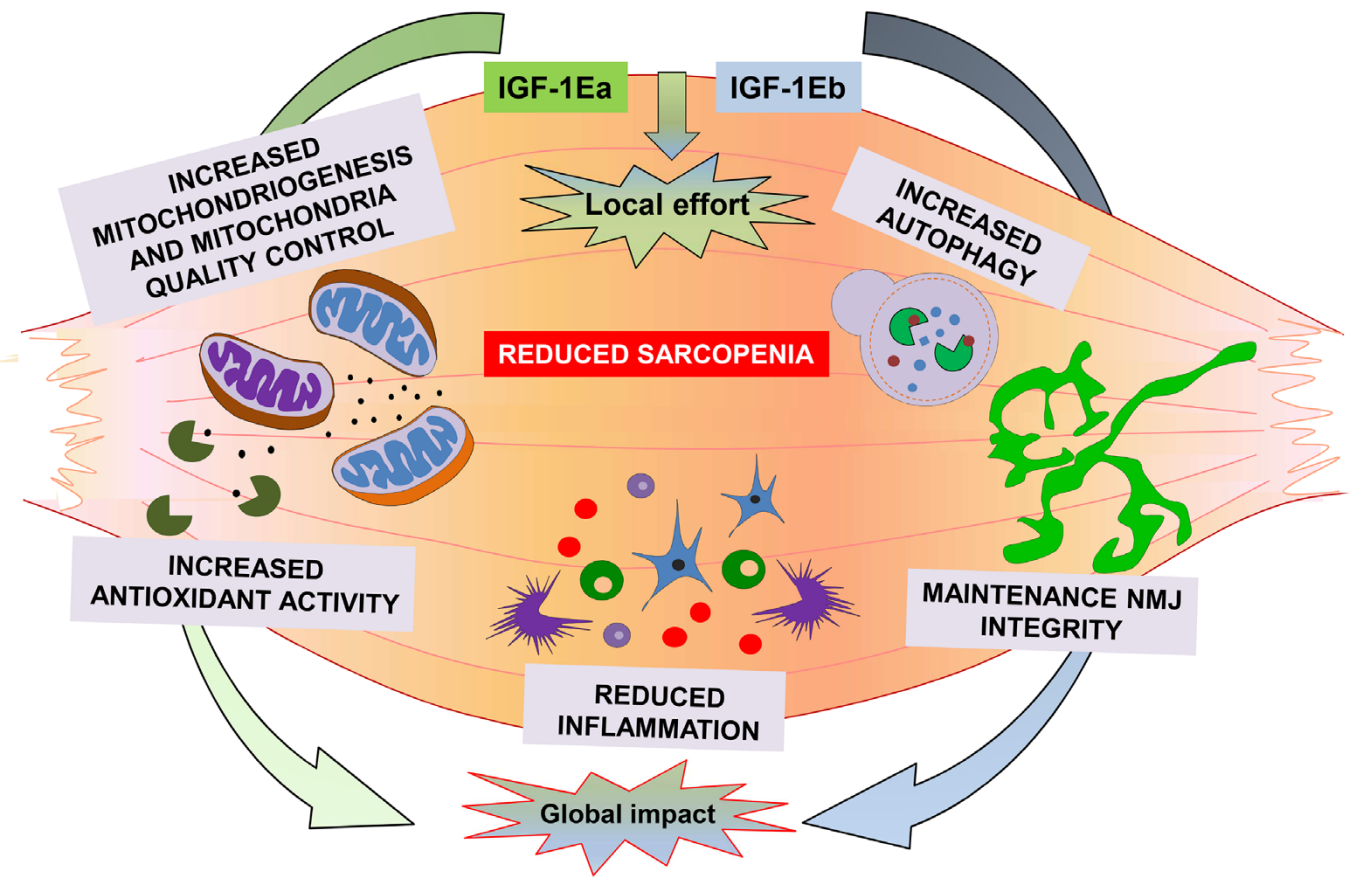
**A summary of the molecular mechanisms activated by both IGF-1Ea and IGF-1Eb to counteract sarcopenia.**

Moreover, the maintenance of hypertrophic phenotype by IGF-1Ea promoted the activation of additional pathways, such as AMPK, a factor involved in the maintenance of whole-body energy balance and an energy sensor controlling glucose and lipid metabolism. These data are consistent with a model (Figure 1) in which muscle expression of either IGF-1Ea or IGF-1Eb, activating a series of anabolic and compensatory pathways, are able to counteract sarcopenia, preventing muscle loss, strength, and alteration in muscle-nerve interaction. It is also plausible that muscle expression of IGF-1 isoforms, preserving skeletal muscle, might maintain the youth not only of muscle tissue but also of the entire organism, by promoting a local effort activity for a global impact benefit.
